# A ReRAM-Based Non-Volatile and Radiation-Hardened Latch Design

**DOI:** 10.3390/mi13111802

**Published:** 2022-10-22

**Authors:** Aibin Yan, Shaojie Wei, Yu Chen, Zhengzheng Fan, Zhengfeng Huang, Jie Cui, Patrick Girard, Xiaoqing Wen

**Affiliations:** 1 School of Computer Science and Technology, Anhui University, Hefei 230601, China; 2 School of Microelectronics, Hefei University of Technology, Hefei 230009, China; 3 Laboratory of Informatics, Robotics and Microelectronics of Montpellier, University of Montpellier, CNRS, 34000 Montpellier, France; 4 Graduate School of Computer Science and Systems Engineering, Kyushu Institute of Technology, Fukuoka 804-8550, Japan

**Keywords:** resistive random-access memory, non-volatile, circuit reliability, latch design, single-event upset

## Abstract

In aerospace environments, high reliability and low power consumption of chips are essential. To greatly reduce power consumption, the latches of a chip need to enter the power down operation. In this operation, employing non-volatile (NV) latches can retain circuit states. Moreover, a latch can be hit by a radiative particle in the aerospace environment, which can cause a severe soft error in the worst case. This paper presents a NV-latch based on resistive random-access memories (ReRAMs) for NV and robust applications. The proposed NV-latch is radiation-hardened with low overhead and can restore values after power down operation. Simulation results demonstrate that the proposed NV-latch can completely provide radiation hardening capability against single-event upsets (SEUs) and can restore values after power down operation. The proposed NV-latch can reduce the number of transistors in the storage cells by 50% on average compared with the other similar solutions.

## 1. Introduction

As power management becomes a critical design concern in VLSI circuits, the non-volatile processing paradigm is becoming more and more popular [1]. Using *Non-Volatile Memory* (*NVM*) to retain the states of circuits in power down operation is a common approach for reducing power dissipation as well as extending the lifetime of a device [2]. Generally, a normal CMOS logic circuit module and a NV storage element (e.g., ReRAM) are extensively used in a NV-latch [3,4]. A soft-error tolerant NV-latch is a critical component of modern electronic devices and can be widely used in aerospace applications [5,6,7].

In the aerospace environment, a chip requires high reliability and low power. The latches in a chip can enter into power down to reduce power consumption. During the power down operation, employing NV-latches can retain circuit states. Moreover, during power-on operation, in the hold mode, a latch can be hit by a radiative particle, which can cause a severe soft error, such as *Single-Event Upset* (*SEU*) [8,9]. Note that, in radiation environments, semiconductor atoms can be ionized by radiative particles (or cosmic rays) to generate electron-hole pairs causing erroneous charges. These charges can be collected by a p-n junction or electrical contact to induce SEUs, which can induce the disturbance of the stored value in a latch during the hold mode [10]. 

However, radiative particles, fortunately, have no obvious effect on ReRAMs because the storage scheme of a ReRAM is based on the formation and elimination of conductive filaments. ReRAM’s storage state changes along with ReRAM’s resistance value. The conductive filaments determine ReRAM’s resistance value, and the more conductive filaments in-side ReRAM, the less resistance ReRAM has. Moreover, the formation of the conductive filaments is controlled by the current of ReRAM. Radiative particles have little effect on the current inside ReRAM that will demonstrate in Section 4. Therefore, radiation particles have no obvious effect on ReRAM. The ReRAM is one of the most promising NVM technologies due to its attractive attributes, such as excellent scalability, low programming voltage, fast switching speed, high endurance against radiation, and compatibility with the CMOS technologies [11]. The advancement of CMOS-compatible ReRAMs has promoted the development of NVMs, which incorporates ReRAMs into CMOS circuits, such as latches, flip-flops, and SRAMs, to provide the NV property. There are many ReRAM-based NVMs, such as *Non-Volatile D-Latch* (*NVDL*) [12], *Radiation-hardened hybrid RRAM-based Non-volatile Latch* (*RHRNL*) [13], *Low Store Energy and Robust Non-Volatile Flip-Flop* (*LSER-NVFF*) [14], *Non-Volatile 8T2R SRAM* (*NV8T2R SRAM*) [15], and *Non-Volatile 7T1R SRAM* (*NV7T1R SRAM*) [16]. However, to the best of our knowledge, there are few latch structures composed of a CMOS logic part along with few ReRAMs to simultaneously provide radiation-hardening and NV capability.

In this paper, a radiation-hardened ReRAM-based NV-latch is proposed. The proposed radiation-hardened NV-latch mainly consists of four *transmission gates* (*TGs*), one *eight-transistor* (*8T*) storage cell, four access transistors (i.e., NMOS transistors), and two ReRAMs. Since the storage cell can form at least one feedback to implement SEU-recovery, the proposed latch is completely immune to SEUs. Due to the features of ReRAMs, the proposed latch can implement NV through value-backup in ReRAMs after power down operation. Moreover, using a small number of transistors in the storage cell, the area consumption of the proposed NV-latch is effectively reduced. Simulation results demonstrate that the proposed NV-latch can restore values through ReRAMs after power down operation and can completely provide radiation hardening capability against SEUs.

The rest of the paper is organized as follows. A brief overview of the ReRAM features, as well as the voltage threshold adaptive memristor (VTEAM) model used to realize the design of the proposed NV-latch are presented in Section 2. Section 3 presents the structure and operation principles of the proposed NV-latch. Simulation and comparison results are introduced in Section 4. Section 5 provides conclusions of this paper. 

## 2. Preliminaries

### 2.1. ReRAM Features

Several transition metal oxides, such as TiO2, HfO2, CuxO, NiO, ZnO, and some perovskite oxides, have a resistance switching feature. A ReRAM, which is made of the above-mentioned resistive material sandwiched between the top and bottom electrodes, can be switched based on the voltage supplied to the electrodes. A ReRAM has two different states: *High Resistance State* (*HRS*) and *Low Resistance State* (*LRS*). The states can be initially attained with a high-voltage forming process (*V_forming_* > *V_set_*) on the original ReRAM. Then, the ReRAM can switch from HRS to LRS when the potential difference between the top and bottom electrodes is *V_set_*. The ReRAM can switch from LRS to HRS when the potential difference between the top and bottom electrodes is *V_reset_*. It is well known that a ReRAM is compatible with the traditional CMOS manufacturing process. The ReRAM also provides a high level of scalability and high performance [17]. 

### 2.2. Voltage ThrEshold Adaptive Memristor (VTEAM) Model for ReRAMs

The VTEAM ReRAM model is widely used to perform hybrid ReRAM/CMOS simulations [18]. The model uses the threshold voltage instead of the threshold current, but internally the threshold is determined by the magnitude of the current. Therefore, the two states of the ReRAM are controlled by the current, and the state of the ReRAM will only change when the current reaches a threshold and maintains it for a period of time. Therefore, the small current or voltage change caused by the quantitative charge will not affect the state of the ReRAM. With a threshold voltage rather than a threshold current, the VTEAM model has the benefits that it is simple, generic, accurate, and designer-friendly. Note that the current-voltage relationship is not inherently defined in the VTEAM model. The VTEAM model is based on an expression, i.e., the derivative of the internal state variable. The derivative of the state variable in the VTEAM model is shown in Equation (1).

(1) $$\frac{dw\left(t\right)}{dt}=\{\begin{array}{ll} k_{set}\cdot {\left(\frac{v\left(t\right)}{v_{set}}-1\right)}^{\alpha_{set}}\cdot f_{set}\left(w\right), & \;0<V_{set}<V\\ 0, & \;V_{reset}<V<V_{set}\\ k_{reset}\cdot {\left(\frac{v\left(t\right)}{v_{reset}}-1\right)}^{\alpha_{reset}}\cdot f_{reset}\left(w\right), & \;V<V_{reset}<0 \end{array}$$

In Equation (1), $k_{set}$, $k_{reset}$, $\alpha_{set}$, and $\alpha_{reset}$ are constants. The parameter $k_{set}$ has a positive value and $k_{reset}$ has a negative value. All of them are fitting parameters. The $V_{set}$ and $V_{reset}$ are threshold voltages. The window functions $f_{set}\left(w\right)$ and $f_{reset}\left(w\right)$ constrain the state variable $w\in \left[w_{reset,\;}\;w_{set}\right]$. The window functions represent the dependence of the derivative of the state variable on w.

## 3. Radiation-Hardened NV-Latch Design and Its Operation Principle

**Figure 1:**
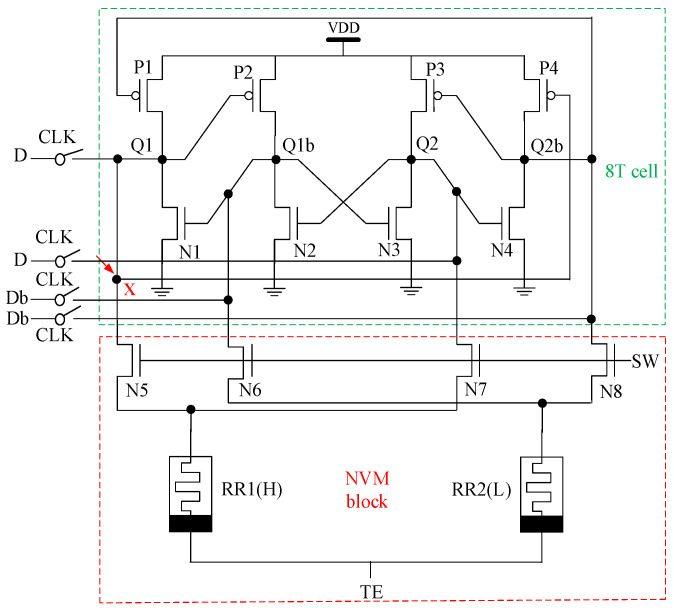
The schematic of the proposed NV-latch.

Figure 1 shows the schematic of the proposed NV-latch based on the VTEAM model described in the previous section. The structure mainly consists of two parts. The first part is the 8T cell. The 8T cell includes two complementary storage pairs, i.e., <Q1, Q1b> and <Q2, Q2b>. The voltages of Q1 and Q1b are identical to Q2 and Q2b, respectively. The second part is the so-called NVM block. The NVM block consists of two ReRAMs along with four access transistors. The NVM block is connected to the 8T cell. 

### 3.1. Operation Principle

**Figure 2:**
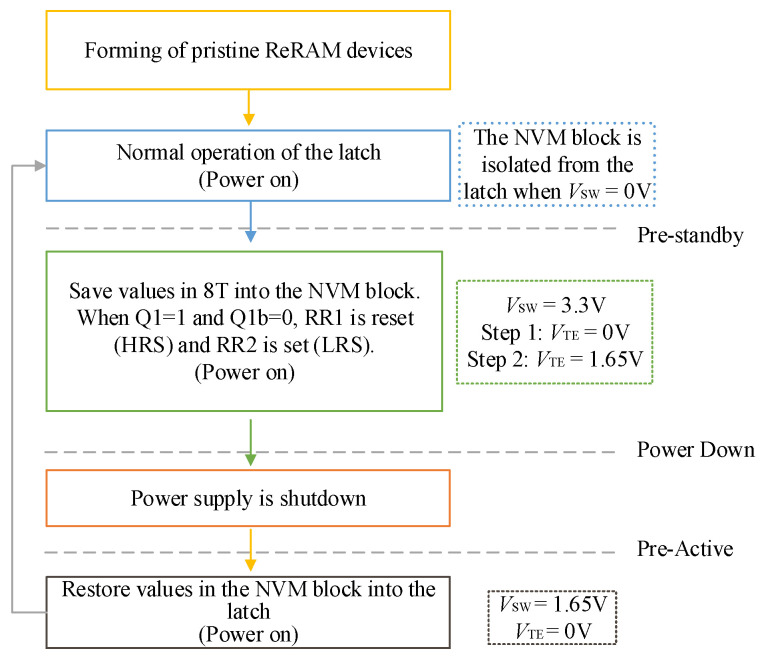
Sequential flow of operations required for transitions between the normal operation and restore operation for the proposed NV-latch.

Figure 2 shows the sequential flow of operations required for transitions between the normal operation and restore operation for the proposed latch. Note that, there are four operations, i.e., normal operation, save operation, power-down operation, and restore operation, for the latch.

#### 3.1.1. Normal Operation

The principles of this operation are the same as a standard latch. The control signal regulating the NVM block is disabled (i.e., $V_{sw}\;$= 0 V) during the operation, resulting in the NVM block being totally isolated from the 8T cell. When CLK = 1, the latch works in transparent mode. As a result, TGs are ON and the values of Q1, Q1b, Q2, and Q2b in the 8T cell are directly driven by D and Db, respectively. When CLK = 0, the latch works in hold mode. At this time, at least one feedback loop can be formed; therefore, the values can be stored in the 8T cell.

#### 3.1.2. Save Operation

The purpose of this operation is to back up the values of the 8T cell into ReRAMs. This operation is controlled by the control signals SW and TE. The 8T cell has two different values (i.e., 0 and 1), and the two independent ReRAMs inside the NVM block require two different polarity biases. There are two steps in the save operation. We discuss the case where the voltage of D is 3.3 V (Q1 = Q2 = 1) and the voltage of Db is 0 V (Q1b = Q2b = 0). Step 1: The values of Q1 and Q2 need to be stored in RR1. At this time, $V_{SW}=3.3$ V, $V_{TE}=0$ V. The potential difference between Q1 and TE can reset RR1 to HRS. Step 2: The values of Q1b and Q2b need to be backed up into RR2. The voltage of SW still remains high whereas TE goes high (see Figure 2). The potential difference between Q1b and TE can set RR2 to LRS. Note that, the high voltage of TE is different from that of the other terminals and it is adjusted to 1.65 V (according to the Equations (2) and (3)) instead of 3.3 V to enhance the error margin of the proposed radiation-hardened NV-latch; Q1, Q1b, Q2, and Q2b, in fact, are unable to achieve the desired high (3.3 V) or low (0 V) voltages. Meanwhile, the access transistors, such as N5, N6, N7, and N8, have conduction resistance. Hence, the voltage values on the bottom electrodes of the ReRAMs are not equal to those of Q1 or Q1b. Note that the voltage of TE should always satisfy Equations (2) and (3). Therefore, the voltage of TE is set to 1.65 V in Step 2.

(2) $$\;\left|V_{TE}-V_{Q1}\right|>\left|V_{reset}\right|$$

(3) $$V_{TE}-V_{Qb1}>V_{set}$$

#### 3.1.3. Power down Operation

The voltage of VDD is reduced throughout the power down operation, and the voltages of all nodes Q1, Q1b, Q2, and Q2b become 0 V, resulting in value loss. The resistance states of ReRAMs, on the other hand, will never diminish during the power down operation.

#### 3.1.4. Restore Operation

During the restore operation, the voltage of the control signal SW should be high for a period of time, which accompanies with the VDD being restored to its original value, to create discharge pathways through ReRAMs to the ground for nodes Q1, Q1b, Q2, and Q2b (the voltage of TE is 0 V). To ensure that the resistance states of ReRAMs will not change, the absolute value of potential difference should be lower than the absolute value of the critical voltage (i.e., $V_{set}$ and $\left|V_{reset}\right|$). SW should be set to a short duration and moderate amplitude so that the access transistors cannot fully open and the channel resistance increases. Thus, the resistance states of these two ReRAMs in discharge pathways cannot be affected by the voltages of nodes Q1, Q1b, Q2, and Q2b.

The values can be restored into the 8T cell due to the resistance difference between the ReRAMs. Note that the pathways having ReRAMs with LRS can discharge much faster than the pathways having ReRAMs with HRS. As a result, the 8T nodes linked to the ReRAMs with LRS will receive a low voltage first and the storage nodes linked to the ReRAMs with HRS will receive a high voltage. Therefore, all values stored in the 8T cell before the power down operation can be restored.

### 3.2. Radiation-Hardening Capability Analysis of The Proposed NV-Latch

In the following, the sufficient radiation-hardening capability in terms of SEUs of the proposed NV-latch is discussed. 

Case 1: To explain the SEU immunity of the 8T cell, the storage state of the cell is set to ‘1010’, which means that values ‘1010’ are stored in Q1, Q1b, Q2, and Q2b, respectively. When a radiative-particle strikes Q1, Q1 tends to be upset to ‘0’. However, Q1b and Q2b are not immediately impacted and remain at ‘0’, thus N1 remains in the OFF state and P1 remains in the ON state. As a result, Q1 continues to be pulled up to VDD and the SEU can be removed. When a radiative-particle strikes Q1b, Q1b tends to be upset to ‘1’. However, Q2b is not immediately impacted and remains ‘0′, thus P3 and P1 remain the ON state. As a result, Q2 remains ‘1’ to keep N2 still being ON. Then, Q1b continues to be pulled downward to GND and the SEU can be removed. If the storage state of the cell is set to ‘0101’, which means that values ‘0101’ are stored in Q1, Q1b, Q2, and Q2b, respectively. When a radiative-particle strikes Q1, Q1 tends to be upset to ‘1’. However, Q1b and Q2b are not immediately impacted and remain at ‘1’, thus N1 remains in the ON state and P1 remains in the OFF state. As a result, Q1 continues to be pulled downward to GND and the SEU can be removed. In fact, if a radiative-particle strikes any other single node, the stored value is always maintained by redundant nodes.

Case 2: The source terminals of the access transistors (i.e., N5, N6, N7, and N8) in the NVM block are the sensitive nodes. Due to the symmetry of the circuit, the source terminal of N5 (node X) in Figure 1 is selected as the sensitive node in the subsequent analysis and simulations. In the save operation, when the storage state of nodes in the latch is set to ‘1010,’ the resistance states of RR1 and RR2 are HRS and LRS, respectively. The values of Q1b, Q2, and Q2b can still be correct when a particle strikes node X during the save operation (refer to the discussions in Case 1). Thus, the resistance state of RR1 can remain HRS. Then, the node-values can still be recovered during the restore operation. Therefore, an SEU can be removed for the proposed NV-latch in this case.

Case 3: When a radiative-particle strikes node X during the restore operation, Q1 will not be affected by the particle. Although the resistance state of RR1 will change because of the particle-striking, there will be no errors in this operation since the value recovery can be accomplished in Case 1. Therefore, the error in ReRAMs cannot be kept during the restore operation.

In summary, the proposed NV-latch is completely SEU hardened in the normal operation. In the save and restore operations, a striking-particle cannot induce errors in the latch. In other words, the proposed NV-latch is completely radiation hardened against SEUs. Therefore, the proposed latch is suitable for robust applications. 

## 4. Simulation and Comparison Results

In all simulations, the VTEAM model was used to be compatible with CMOS, and the values of $V_{set}$ and $V_{reset}$ were set to 0.45 V and −1 V, respectively. The resistances of LRS and HRS were set to 1 kΩ and 100 kΩ, respectively. In detail, a mature CMOS design kit for peripheral circuits and the VTEAM model for the ReRAM were used to show the radiation-hardening capabilities and performance of the proposed NV-latch. Radiation-induced transient current (SEU) was simulated and a double exponential current source was linked to sensitive nodes to effectively and precisely characterize this type of transient current [19,20,21]. The double exponential current source is presented in Equation (4).

(4) $$I\left(t\right)=\frac{Q_{inj}}{\tau_{1}-\tau_{2}}\left(e^{-\frac{1}{\tau_{1}}}-e^{-\frac{1}{\tau_{2}}}\right)$$

In Equation (4), $Q_{inj}\;$ is the amount of the injected charge, which is related to the linear energy transfer of a particle, $\tau_{1}$ is the collection time constant of the junction, and $\tau_{2}$ is the time constant for the initial establishment of the ion track. In this work, $\tau_{1}$ and $\tau_{2}\;$ were respectively set to 5 ns and 500 ps that are large enough, while $Q_{inj}$ was set to 100 fC to mimic the extreme disturbance of SEUs. Then, extensive simulations using Synopsys HSPICE tool were performed.

**Figure 3:**
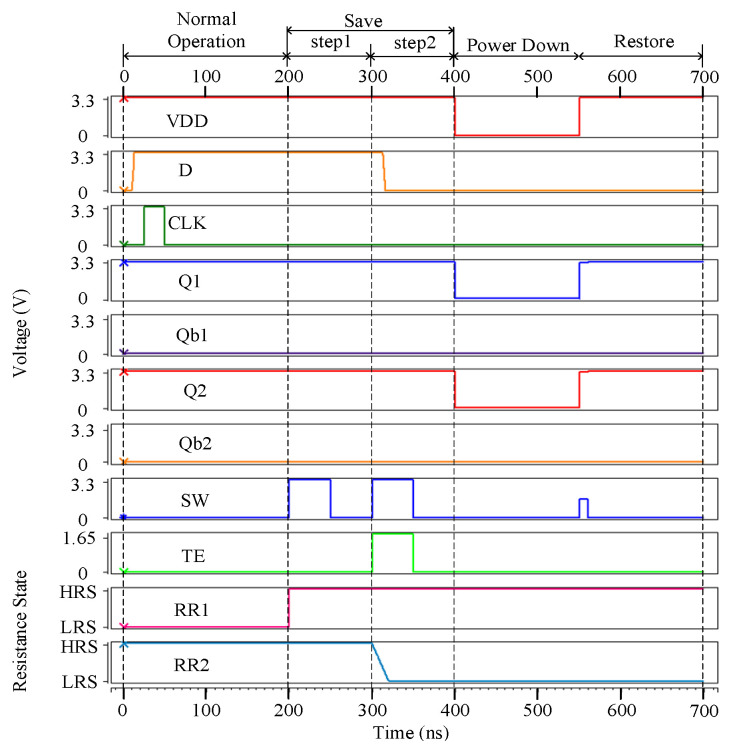
Error-free simulation result for the proposed NV-latch.

Figure 3 shows the error-free simulation result for the proposed NV-latch, which proves the value recovery capability of the latch. The original values stored in Q1, Q1b, Q2, and Q2b were set to ‘1’, ‘0’, ‘1’, ‘0’, respectively. It can be seen from Figure 3 that, during the normal operation, the working principles of the proposed NV-latch are similar to those of a standard latch. In the save operation, according to the values of Q1, Q1b, Q2, and Q2b, the resistance states of ReRAMs were set to HRS and LRS, respectively. During the power down operation, the voltage of VDD became 0 V. During the restore operation, the values were restored according to the resistance states of ReRAMs. Therefore, the error-free operations of the proposed NV-latch are correct. 

**Figure 4:**
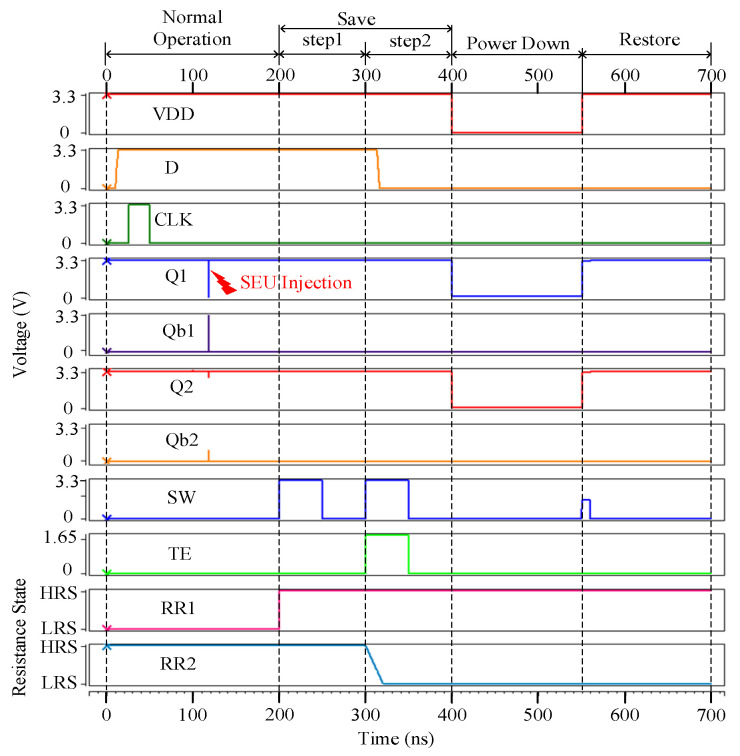
SEU injection into node Q1 of the proposed NV-latch during the normal operation.

Figure 4 shows the case that node Q1 suffers from an SEU for the proposed NV-latch during the normal operation. As for the simulation of Case 1 considering one node inside the 8T cell, Q1 was selected for the SEU injection simulation. As shown in Figure 4, at 120 ns, an SEU was injected on Q1. It can be seen from Figure 4 that the proposed NV-latch is effectively radiation-hardened (self-recoverable from the injected SEU) for Case 1.

**Figure 5:**
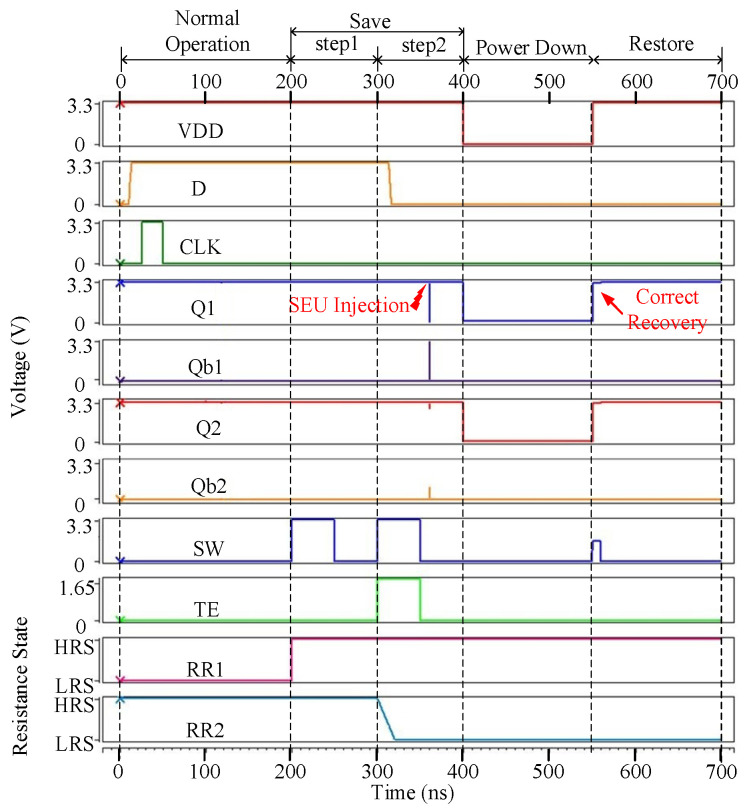
SEU injection into node X of the proposed NV-latch during the save operation (step 2).

Figure 5 shows the injected SEU on node X of the proposed NV-latch during the save operation (see the above-mentioned Step 2). As mentioned above, Case 2 includes presentative node X. During Step 2 in the save operation, a radiative particle struck node X at 350 ns, as shown in Figure 5, and the resistance state of the RR1 still remained HRS. Then, during the restore operation, Q1 was restored to ‘1’ at 550 ns, which is the correct value initially stored in Q1. This is owing to the interlocked feedback mechanism used in the 8T cell. Therefore, the proposed NV-latch is effectively radiation-hardened for Case 2.

**Figure 6:**
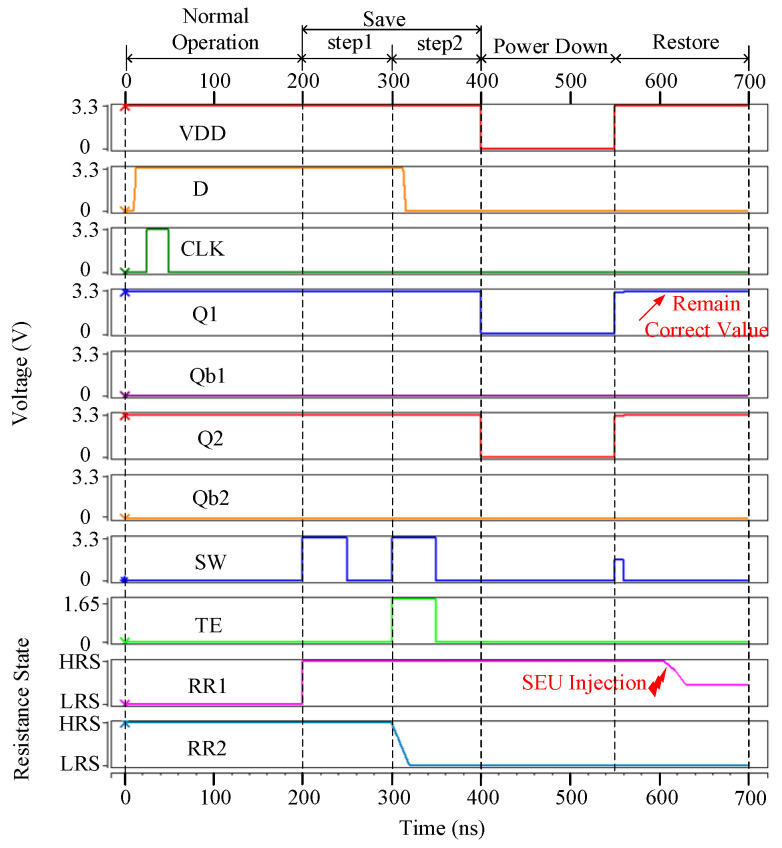
SEU injection into node X in the proposed NV-latch during the restore operation.

Figure 6 shows the simulated SEU on node X in the proposed NV-latch during the restore operation. It can be seen that, the value of Q1 remained correct when a radiative particle struck node X at 600 ns during the restore operation, even though the resistance state of RR1 was about to change. The error cannot be kept because the recovery of values was completed and the NVM block was isolated from the 8T cell by the voltage of SW that is 0 V, as shown in Figure 6. Therefore, the proposed NV-latch is effectively radiation-hardened for Case 3.

The above-mentioned simulation results show that the proposed NV-latch is immune to the influence of SEUs. In detail, the injection of high-energy particles in the normal operation and the save operation cannot induce retained errors, because of the redundant storage nodes and interlocking feedback mechanism in the 8T cell of the proposed NV-latch. Meanwhile, the SEU injection in the restore operation also cannot induce retained errors because of the control signal SW. In summary, the proposed NV-latch can not only restore correct values through ReRAMs after the power down operation, but also provide radiation-hardening capability throughout the various operations.

Moreover, the state (i.e., set and reset) of ReRAM does not transition when an SEU was injected into the proposed NV-Latch. In the simulation, the amount of charge injected into the circuit is fixed. Since Q*_inj_* = UIT (Q*_inj_* refers to the amount of charge, U is voltage, I is current, and T is time), when the voltage of the injected SEU reaches 3.3 V, the change of current is small. Therefore, it will not affect the state of ReRAM.

To the best of our knowledge, there are few latch structures composed of a CMOS logic part along with few ReRAMs to simultaneously provide radiation-hardening and NV capability. To make a fair comparison, the traditional unhardened latch, *Triple-Module-Redundancy based Latch* (*TMRL*), *Low-power Soft Error Hardened latch* (*LSEH*) [22], *Feedback Redundant Soft Error Tolerant latch* (*FERST*) [23], *Circuit and Layout Combination Technique based latch* (*CLCT*) [24], *High-Performance Robust latch* (*HiPeR*) [25], and *Low Power and High Speed latch* (*LPHS*) [26] were selected as storage cells to implement NV-latches. These above latches and RHRNL were implemented/designed under the same conditions as that of the proposed NV-latch. 

Table 1 shows the comparisons of NV-latches. The unhardened storage module in a NV-latch has the worst tolerance against soft errors (i.e., injected-charge-induced SEUs) with only a measured critical-charge of about 5.13 fC (see Table 1). The storage modules, such as TMRL, LSEH, FERST, CLCT, HiPeR and LPHS, and the storage modules in the RHRNL and in the proposed latch have relatively better radiation-hardening performance. The critical charge of TMRL is about 6.21 fC, while the critical charge of LSEH is 6.39 fC. The storage modules, such as TMRL, LSEH, FERST, CLCT, HiPeR and LPHS, and the storage modules in the RHRNL and in the proposed latch have an identical ‘∞’ critical charge, which means that they can tolerate/remove any SEU regardless the energy level of the striking particles. 

It can be seen from Table 1 that, the number of the consumed CMOS transistors in the proposed NV-latch is large than that of the unhardened-latch-based NV-latch mainly due to the use of eight transistors in the storage module of the proposed NV-latch to ensure high reliability. The proposed NV-latch has extra transistors as compared to the unhardened-latch-based NV-latch which only contains four transistors in the storage module. However, the proposed latch consumes less CMOS transistors than the other NV-latches, such as TMRL, LSEH, FERST, CLCT, HiPeR, and LPHS based ones that have more than eight transistors in the storage module. Although the proposed NV-latch use the same number of transistors as RHRNL in the storage cell, the proposed NV-latch only use two ReRAMs. 

Table 2 shows the detailed cost comparisons of NV-Latches based on different storage cells. The cost analysis metrics for comparisons include delay, area, power, and Delay-Area-Power Product (DAPP). The delay refers to the delay from D to Q, i.e., the average of the rise and fall delays from D to Q. The area refers to the total area of all CMOS transistors in latches [27]. The power consumption denotes the average of power dissipation (dynamic and static). The DAPP metric is calculated by multiplying delay, area, and power. According to Table 2, it can be calculated that, compared with all the reference NV-latches, the proposed NV-latch can reduce DAPP by 99.50%, delay by 91.83%, area by 62.11% and power consumption by 46.72% on average. 

As can be seen from Table 2, the design in [13] has small overhead than the design in this paper. The reason is that we fairly only consider the CMOS part for overhead evaluation. The overhead of ReRAM and CMOS depends on different parameters (such as area, resistance value, etc.) of their model. The library of CMOS and ReRAM is different, and simply adding their overhead is not fair. The proposed design contains two ReRAM cells, and the design in [13] contains four ReRAM cells. Therefore, when the model parameters of ReRAM are large, the design overhead in [13] is large than that of the proposed design. In order to quantitatively compare the design in [13] with the proposed design in this paper, we use ReRAM with 90 nm × 90 nm process and CMOS transistor with 22 nm process for evaluations of these two designs. Table 3 shows the cost comparison between the design in [13] and the proposed design in this paper. It can be seen from Table 3 that, under the same simulation conditions, the design in this paper is superior to the design in [13] in terms of area, power consumption and delay. In summary, the proposed NV-latch is a promising radiation-hardened structure for use in robust and low-cost applications.

## 5. Conclusions

With the advancement of semiconductor technologies, power management of circuits and systems has become a vital concern. A NV-latch, which can save values even after the power down operation, can be a good solution for power problems. Moreover, the latch can be affected by radiation in the aerospace environment, which affects the reliability of circuit states. In this paper, a radiation-hardened ReRAM based NV-latch with an 8T cell structure has been proposed. Using a small number of transistors, the latch has low overhead in terms of area. According to pertinent simulation results, the proposed radiation-hardened NV-latch can recover values from ReRAMs. Moreover, it completely provides radiation hardening capability against SEUs induced by the injected charge. In terms of the number of transistors in the storage cells, the proposed radiation-hardened NV-latch can reduce by 50% on average compared with the other similar radiation-hardening solutions. Therefore, the proposed NV-latch is suitable for robust applications.

## Figures and Tables

**Table 1:** Comparisons of NV-latches based on different storage cells.

Storage Cells of NV-Latches	Critical Charge to Induce an SEU (fC)	Transistors in the Storage Cells	ReRAMs in the NVM Block
Unhardened	5.13	4	2
TMRL	6.21	24	4
LSEH [22]	6.39	18	3
FERST [23]	∞	16	3
CLCT [24]	∞	24	4
HiPeR [25]	∞	17	3
LPHS [26]	∞	18	3
RHRNL [13]	∞	8	4
Proposed	∞	8	2

**Table 2:** Detailed Cost Comparisons of NV-Latches Based on Different Storage Cells.

Storage Cells of NV-Latches	Delay (ps)	10^−4^ × Area (nm^2^)	Power (uw)	DAPP
TMRL	37.80	6.76	7.06	1804.03
LSEH [22]	7.51	3.66	0.94	25.84
FERST [23]	7.05	3.94	1.53	42.50
CLCT [24]	35.60	4.51	1.42	228.00
HiPeR [25]	0.60	2.25	1.23	1.66
LPHS [26]	3.49	3.38	0.72	8.49
RHRNL [13]	1.29	0.81	0.50	0.52
Proposed	1.09	1.37	1.02	1.52

For fair comparisons, only the overhead of CMOS part is considered. Note that the RHRNL has four ReRAMs but the proposed has only two ReRAMs.

**Table 3:** The Cost Comparison between the Design in [13] and the Proposed Design.

NV-Latches	Delay (ps)	10^−4^ × Area (nm^2^)	Power (uw)	DAPP
RHRNL [13]	1.29	4.05	73.41	383.53
Proposed	1.09	2.99	15.39	50.16

## Data Availability

Not applicable.
